# Molecular Mechanisms of Leucine Zipper EF-Hand Containing Transmembrane Protein-1 Function in Health and Disease

**DOI:** 10.3390/ijms20020286

**Published:** 2019-01-12

**Authors:** Qi-Tong Lin, Peter B. Stathopulos

**Affiliations:** Department of Physiology and Pharmacology, Schulich School of Medicine and Dentistry, The University of Western Ontario, London, ON N6A 5C1, Canada; qlin44@uwo.ca

**Keywords:** leucine zipper, EF-hand, coiled-coil, LETM1, LETM2, calcium signaling, Na^+^/K^+^ exchanger, Ca^2+^/H^+^ exchanger, structural mechanism, Wolf-Hirschhorn syndrome, cancer, mitochondria

## Abstract

Mitochondrial calcium (Ca^2+^) uptake shapes cytosolic Ca^2+^ signals involved in countless cellular processes and more directly regulates numerous mitochondrial functions including ATP production, autophagy and apoptosis. Given the intimate link to both life and death processes, it is imperative that mitochondria tightly regulate intramitochondrial Ca^2+^ levels with a high degree of precision. Among the Ca^2+^ handling tools of mitochondria, the leucine zipper EF-hand containing transmembrane protein-1 (LETM1) is a transporter protein localized to the inner mitochondrial membrane shown to constitute a Ca^2+^/H^+^ exchanger activity. The significance of LETM1 to mitochondrial Ca^2+^ regulation is evident from Wolf-Hirschhorn syndrome patients that harbor a haplodeficiency in LETM1 expression, leading to dysfunctional mitochondrial Ca^2+^ handling and from numerous types of cancer cells that show an upregulation of LETM1 expression. Despite the significance of LETM1 to cell physiology and pathophysiology, the molecular mechanisms of LETM1 function remain poorly defined. In this review, we aim to provide an overview of the current understanding of LETM1 structure and function and pinpoint the knowledge gaps that need to be filled in order to unravel the underlying mechanistic basis for LETM1 function.

## 1. Introduction

Calcium (Ca^2+^) is a divalent cation that regulates many facets of cell physiology such as gene expression, muscular contraction, fertilization, proliferation and bioenergetics, to name a few. Eukaryotic cells have evolved to use Ca^2+^ not only as a primary messenger when binding and activating or inactivating enzymes and proteins, but also as a secondary messenger involved in the signal transduction of various pathways. Thus, Ca^2+^ signaling drives a spectrum of cellular events from cell growth and proliferation to cell cycle arrest and apoptosis [1]. The concentration of intracellular Ca^2+^ in the cytosol is typically maintained within ~50–100 nM, much lower than extracellular Ca^2+^ levels that lie within approximately the mM range. As a result, Ca^2+^ is being constantly pumped out of the cytosol, through the largely ion-impermeable plasma membrane using primary pumps such as the plasma membrane Ca^2+^ ATPase (PMCA) and secondary transporters such as the sodium (Na^+^)/Ca^2+^ exchanger [2,3,4].

Due to the wide-ranging effects that changes in Ca^2+^ concentrations can have inside cells, the fine control of Ca^2+^ flux and maintenance of homeostasis from extracellular and intracellular Ca^2+^ stores are critical for the ability of cells to normally function. Intracellular Ca^2+^ demanded by cell signaling can be supplied through an influx of Ca^2+^ from extracellular free Ca^2+^ driven by a 10,000-fold inward gradient, or released into the cytosol from the endoplasmic/sarcoplasmic reticulum (ER/SR) internal Ca^2+^ stores [3,4,5]. The release of intracellular ER/SR-stored Ca^2+^ can be mediated, for example, by inositol 1,4,5-trisphosphate receptor (IP_3_R) channels that are activated by inositol 1,4,5-trisphosphate or ryanodine receptor channels that can be activated by multiple different stimuli [6,7,8,9,10]. Because internal ER stores of Ca^2+^ are maintained at ~400–800 μM, which is ~400–1000-fold higher than the cytosol, IP_3_R activation results in a quick increase in cytosolic Ca^2+^ levels. However, this internal Ca^2+^ store of the ER is limited and exhaustible; therefore, for signaling events requiring more prolonged elevation of cytosolic Ca^2+^, the activation of store operated Ca^2+^ entry (SOCE) occurs in response to ER Ca^2+^ store depletion [5,11]. SOCE is primarily driven by the formation and gating of Ca^2+^ release-activated Ca^2+^ (CRAC) channels composed of Orai1 subunits on the plasma membrane. The activation of these CRAC channels is mediated by stromal interaction molecule-1 and -2 (STIM1/2) which function as ER luminal Ca^2+^ sensors that directly couple to Orai1 subunits and mediate gating of the channel after detecting decreases in ER Ca^2+^ levels [12,13,14,15,16]. Ultimately, the increased cytosolic Ca^2+^ mediated by SOCE not only signals downstream processes, but also replenishes the ER Ca^2+^ stores via the action of the sarcoplasmic/endoplasmic Ca^2+^ ATPase (SERCA) pumps [17].

While initially thought to be the primary intracellular Ca^2+^ store prior to the identification of the ER as the major intracellular store, today the mitochondrion has been well-established as a Ca^2+^ uptake organelle vital for signal transduction and enzyme activation as well as an important Ca^2+^ sink when chelated with inorganic phosphate [18,19,20,21,22,23,24,25,26,27]. The uptake of cations such as Ca^2+^ and potassium (K^+^) (see below) into mitochondria is driven by the large negative membrane potential across the inner mitochondrial membrane (IMM) (i.e., ~−180 mV in the matrix relative to the intermembrane space (IMS)) [28,29,30]. The negative IMM potential is established by proton pumping from the matrix into the IMS by the respiratory chain complexes [27,31,32]. The movement of protons down the electrochemical gradient back into the matrix promotes ATP synthase-mediated generation of ATP. Within the matrix, increased Ca^2+^ can enhance the activity of dehydrogenases, which increase the levels of nicotinamide adenine dinucleotide (NADH) [33,34,35,36]. Augmented NADH levels promote respiratory chain activity and ATP generation [37]. However, chronically elevated intramitochondrial (i.e., matrix) Ca^2+^ can stimulate futile Ca^2+^ cycling involving Na^+^/Ca^2+^ and Na^+^/H^+^ exchangers, ultimately compromising the large negative membrane potential due to the extrusion of matrix Na^+^ in exchange for H^+^ [27,38]. More severe intramitochondrial Ca^2+^ overload can lead to mitochondrial permeability transition pore (mPTP) opening. The mPTP is a large voltage independent, non-specific channel of the IMM which facilitates the release of mitochondrial solutes of up to ~1.5 kDa. Besides Ca^2+^ overload in the matrix, mPTP opening can be instigated by oxidative damage [39]. The full molecular identity of the mPTP remains unresolved; however, several proteins are suggested to be important for the function of mPTP including peptidyl-prolyl isomerase F, the ADP/ATP translocase, the F1-FO-ATP synthase, spastic paraplegia 7 and mitochondrial calcium uniporter regulator-1 (MCUR1) [39,40,41,42,43,44]. The mPTP-induced non-selective flow of ions and small molecules in both directions increase osmotic pressure and further decreases the membrane potential, leading to mitochondria swelling, disruption in oxidative phosphorylation, and ultimately driving cell death [43,45,46]. Thus, the proper regulation and maintenance of ion concentrations in mitochondria is crucial for normal cell function and survival. 

The outer mitochondrial membrane (OMM) is quite permeable to most ions and small molecules (i.e., <5 kDa), owing to the relatively porous function of voltage dependent anion channels (VDAC). However, the IMM exhibits much higher ion selectivity due to the localization and function of specific protein machinery. The key players mediating Ca^2+^ uptake and homeostasis within mitochondria remained elusive until relatively recently. In 2011, the major pore forming subunit of the mitochondrial calcium uniporter (MCU) was identified [47,48]. MCU facilitates highly Ca^2+^ selective movement across the IMM without the exchange of another ion [49]. MCU functions as a hetero-complex in an association of many proteins including the MCU pore forming unit, regulatory mitochondrial calcium uptake (MICU1-3), essential MCU regulator (EMRE), MCUR1, MCUb and solute carrier 25A23 (SLC25A23) [39,47,48,50,51,52,53]. Structural studies have revealed that the MCU pore is a tetramer and contains a soluble N-terminal domain within the matrix. The pore forming, C-terminal region of the protein contains a well-conserved WDXXEPVTY motif (shown in single letter amino acid code where X denotes hydrophobic amino acids) which mediates the high Ca^2+^ selectivity. Approximately 10,000 Ca^2+^ ions can pass through this selectivity filter per second. Interestingly, the N-terminal matrix domain contains a divalent cation binding site that regulates MCU architecture and function, in a feedback-like mechanism observed for many other Ca^2+^ channels [54,55]. 

Clearly, uptake of Ca^2+^ into the matrix without a balancing efflux could be catastrophic due to osmotic swelling, effects on bioenergetics and activation cell death pathways. A major Ca^2+^ efflux pathway is the Na^+^/Ca^2+^ exchanger which can utilize either the Na^+^ or lithium (Li^+^) (NCLX) gradient to remove Ca^2+^ from the matrix [56,57]. NCLX is expressed on the IMM in all tissues, similar to MCU. Topologically, NCLX has two domains each with six transmembrane segments. NCLX transports 1 Ca^2+^ out of the matrix for 3 Na^+^ from the IMS which is equilibrated with the cytosol. 

Importantly, the leucine zipper EF-hand containing transmembrane protein-1 (LETM1) functions as Ca^2+^/H^+^ exchanger and plays a vital role in maintaining Ca^2+^ homeostasis within the matrix [58,59,60,61,62,63,64]. A K^+^/H^+^ exchange (KHE) activity was first characterized for LETM1, and, today, the monovalent cation exchange remains a debated function for this protein. The importance of LETM1 to mitochondrial cation homeostasis is well-established from studies linking LETM1 expression, function and post-translational modifications to numerous pathophysiologies. Nevertheless, despite the significance of LETM1 to health and disease, insights into the molecular mechanisms of LETM1 function remain limited. In this review, we aim to provide an overview of the current understanding of LETM1 structure and function, and identify important new research questions which are needed to unravel the underlying mechanistic basis for LETM1 function. 

## 2. LETM1 Protein Family Domain Architecture and Homology

### 2.1. The LETM1 Protein Family

**Figure 1:**
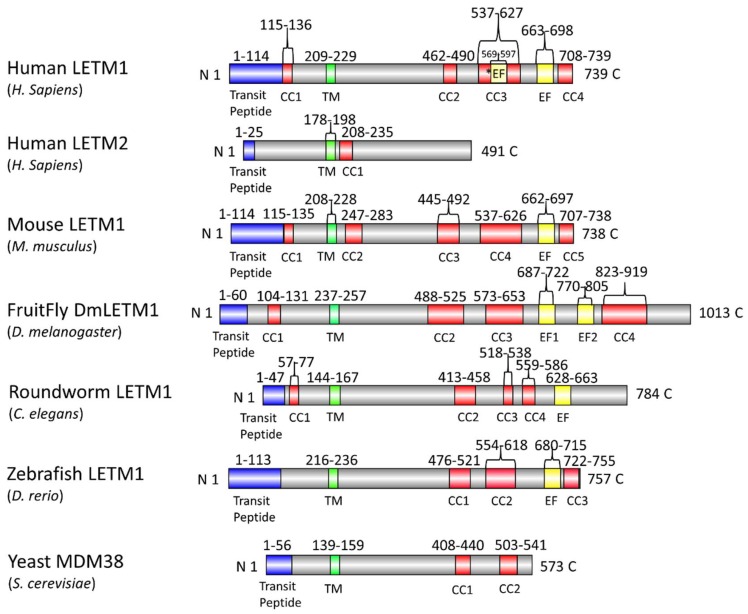
Predicted domain architecture of full length leucine zipper EF-hand containing transmembrane protein-1 (LETM1) orthologs in higher and lower order organisms. The N-terminal regions (upstream of the transmembrane domain) reside within the mitochondrial intermembrane space (IMS). The C-terminal regions (downstream of the transmembrane domain) reside in the mitochondrial matrix. Coiled-coils are denoted with CCn (red; where n identifies sequential domain numbers), EF-hand motifs are denoted with EFn (yellow; where *EF denotes the putative non-canonical EF-hand motif reported in the literature [65,70]), and transmembrane domains are denoted with TM (green). The regions corresponding to the transit peptides are shaded blue. Numbers above each domain correspond to the residue ranges defining each domain. The amino and carboxyl termini are denoted by N and C, respectively. The relative location of each domain is based on the UniProt annotations. The UniProt accession numbers for the *D. rerio* (zebrafish), *M. musculus* (mouse), *D. melanogaster* (fruitfly), *C. elegans* (roundworm), *S. cerevisae* (yeast) and *H. sapiens* (human) LETM1 orthologs are Q1LY46, Q9Z2I0, P91927, Q9XVM0, Q08179 and O95202, respectively. The UniProt accession number for the *H. sapiens* (human) LETM2 protein is Q2VYF4.

In humans, the LETM1 protein family consists of LETM1 encoded on chromosome 4p16.3 and a LETM1-like protein, LETM2, found on chromosome 8p11.2 [65,66,67]. LETM1 is the focus of this manuscript and is discussed in detail in the sections below. LETM2 was initially discovered in a small region within chromosome 8p11.2 immediately adjacent to the Wolf-Hirschhorn syndrome candidate-1-like gene-1 (*WHSC1L1*); *WHSC1L1* is highly homologous to the Wolf-Hirschhorn syndrome candidate-1 (*WHSC1*) gene found in chromosome 4p13.3, which is deleted in patients suffering from Wolf-Hirschhorn syndrome (WHS) [67]. The region adjacent to *WHSC1L1* was found to encode a LETM1-like protein which exhibits a high sequence similarity (i.e., ~52%) to human LETM1, corresponding to the region between the transmembrane domain and putative leucine-zipper coiled-coil domain [62]. Remaining largely uncharacterized, LETM2 contains a putative leucine zipper coiled coil domain (i.e., between residues 208–235), but lacks the putative EF-hand motif and is significantly smaller than LETM1. Specifically, the LETM2 protein is predicted to be 491 residues compared to human LETM1, which is made up of 739 residues (Figure 1). In a study where LETM1 and LETM2 expression was assessed in rat tissues, it was found that, while LETM1 was ubiquitously expressed, LETM2 was only highly expressed in the testis and sperm [62]. In terms of conservation in lower order organisms, both LETM1 and LETM2 homologs are found to be expressed in lower eukaryotes, bacteria and protozoa [62,68,69,70].

### 2.2. LETM1 Domain Architecture and Homology

LETM1 is a single transmembrane domain protein that has been shown to transport ions across the IMM. It is synthesized by cytosolic ribosomes as an 83.4 kDa protein which is then transported to the IMM as a 70 kDa protein upon cleavage of the signal peptide (~13.4 kDa) [62]. While both KHE and Ca^2+^/H^+^ exchange (CHE) activity have been proposed, it is possible LETM1 performs both activities either directly or through indirect means. Indirectly, it has been proposed that the Ca^2+^ flux activity of LETM1 may stabilize or activate K^+^ transporters in the IMM [62]. Nevertheless, the prevailing consensus in the field is that the primary functional role of LETM1 is to mediate CHE activity across the IMM [58,59,60,61,62,63,64]. Indeed, LETM1 is localized in the IMM via a single transmembrane domain [61,62,70]. 

**Figure 2:**
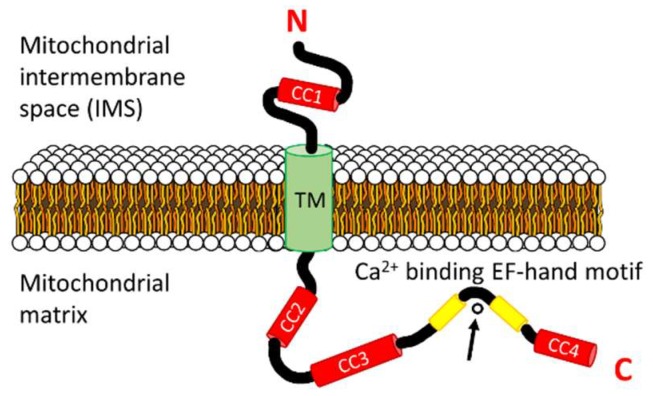
Prevailing topology of the full length human LETM1 monomer. The topology is shown with respect to the inner-mitochondrial membrane (IMM) showing the N-terminal region within the mitochondrial inter-membrane space (IMS) and the C-terminal region within the mitochondrial matrix. The locations of the coiled-coils (CCn; red) relative to the transmembrane domain (TM; green), the EF-hand motif (yellow) and the IMS and matrix compartments are shown. The Ca^2+^ cation coordinated by the EF-hand loop is indicated by the arrow. The amino and carboxyl termini are denoted by N and C, respectively.

Human LETM1 has a hydrophobic N-terminal domain (i.e., residues 115–208) oriented in the mitochondrial IMS [61]. This N-terminal region contains a putative coiled coil domain prefaced by a signal peptide (i.e., signal peptide: residues 1–114; coiled coil 1: residues 115–136). A single transmembrane domain spanning the IMM (i.e., residues 209–229) separates the N-terminal domain from the larger and hydrophilic C-terminal domain (i.e., residues 230–739) which is localized in the mitochondrial matrix [59,61]. The matrix-oriented C-terminal region contains a putative canonical EF-hand motif (i.e., residues 663–698) and three predicted coiled coil domains (i.e., coiled coil 2: residues 462–490; coiled coil 3: 537–627; coiled coil 4: 708–739) (Figure 2). A second, non-canonical EF-hand motif has been suggested to exist (i.e., residues 569–597) within the C-terminal domain of human LETM1, proximal to the transmembrane region [65,70]. However, this second, non-canonical EF-hand is embedded in the region predicted to form a coiled coil 3 (Figure 1). 

EF-hand motifs are helix-loop-helix structures that coordinate Ca^2+^ in the 12-residue loop [71,72]. There are hundreds of EF-hand protein family members in humans, and residue conservation within the loop allows for the prediction of canonical Ca^2+^ coordination with pentagonal bipyramidal geometry [71,72,73]. Residue positions 1, 3, 5, 7 and 12 directly orient side chain or backbone oxygen atoms, while position 9 often bridges a water molecule oxygen atom in the Ca^2+^ coordination [73]. A largely invariant Gly at position 6 gives canonical EF-hand loops the conformational freedom required for tight Ca^2+^ binding involving 7 oxygen atoms (note that position 12 provides two side chain oxygen atoms) [71,73,74]. The LETM1 putative canonical EF-hand loop contains prevalent Ca^2+^ coordinating residues at positions 1, 3, 5, 7, 9 and 12 and has the important Gly at position 6. The most unconventional aspect of the LETM1 canonical loop is the Asp at position 12 which tends to be Glu in most EF-hand proteins; however, Asp is the second most prevalent residue at position 12 among EF-hand protein family members [73]. The Asp at this position likely weakens the Ca^2+^ binding affinity, perhaps due to the shorter side chain and less than ideal coordination geometry within the EF-hand loop. Herein, we have termed the second LETM1 EF-hand as “non-canonical” because the loop lacks the invariant Gly at position 6 and prevalent Ca^2+^ coordinating residues at position 3 and 9 [73]. EF-hand motifs are usually found in pairs that are stabilized by mutual hydrogen bonding between loops [71,74]. The pairing endows these EF-hand domains with a cooperativity in Ca^2+^ binding and structural response [71,73,74]. STIM1 contains a canonical Ca^2+^ binding EF-hand paired with a non-canonical EF-hand motif devoid of Ca^2+^ binding [75]. The non-canonical EF-hand plays an integral structural role in the Ca^2+^ sensing mechanism of STIM1 [76]. The amino acid sequence of the LETM1 non-canonical EF-hand motif identified in past studies [65,70] is not similar to the non-canonical EF-hand motifs of higher or lower order STIM proteins (i.e., human, fruitfly, roundworm, and chicken). Thus, it remains to be confirmed by high-resolution structural elucidation whether the canonical EF-hand motif is paired in the regulation of LETM1 function.

**Figure 3:**
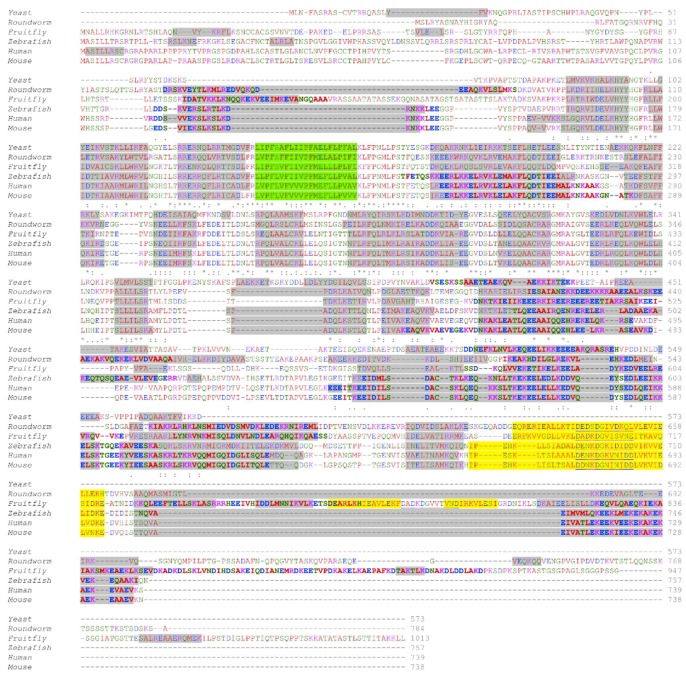
Primary structure alignment of LETM1 orthologs from higher and lower order organisms and predicted secondary structure and coiled-coil elements. The amino acid sequence alignment was performed using Clustal Omega [78] on LETM1 orthologs across six different species. Residue colors represent amino residue characteristics as follows: acidic (blue), basic (magenta), polar/uncharged (green) and hydrophobic (red). Residue conservation is shown below the alignment as fully conserved (*), highly conserved (:) or partially conserved (.). The locations of predicted α-helices using PSIPRED [79] are shaded grey. Green and yellow shading indicate the locations of the conserved transmembrane domains and EF-hand helices, respectively, based on the UniProt annotations. Underlining (blue) residues between the EF-hand helices indicate the location of the canonical 12 residue Ca^2+^ binding loops. The prediction of the coiled-coils was performed using the Coiled-Coil Prediction server [80], using a 21 residue window, shown in bolded font. The UniProt accession numbers for the yeast, roundworm, fruitfly, zebrafish, human and mouse LETM1 proteins used in this alignment and annotation are Q08179, Q9XVM0, P91927, Q1LY46, O95202 and Q9Z2I0, respectively.

LETM1 homologs from lower and higher order organisms share different aspects of the basic domain architecture. Yeast MDM38 and MRS7 proteins share similar primary structure and domain topology, particularly within the single transmembrane domain, and are localized to the IMM. Deletion of *MDM38* has been shown to cause swelling of mitochondria and dissolution of well-defined mitochondria cristae structure [70,77]. Remarkably, MDM38 shares a ~42% amino acid sequence similarity with human LETM1; however, MDM38 lacks the canonical EF-hand at the far C-terminal region (Figure 1 and Figure 3). Thus, the MDM38 domain architecture resembles that of human LETM2 to a greater degree than LETM1 [69]. 

*Drosophila melanogaster* LETM1 (DmLETM1) is composed of over 1000 residues (i.e., 1013 residues). Unlike the other orthologs, *D. melanogaster* LETM1 clearly contains two putative canonical EF-hand motifs which are identifiable based on amino acid sequence. A global amino acid sequence alignment of *D. melanogaster* LETM1 with human LETM1 using Clustal Omega [78] shows the proteins have similar N-terminal and C-terminal domains, with the *Drosophila* LETM1 extended at the far C-terminal region compared to the human ortholog. Interestingly, the first EF-hand of *Drosophila* LETM1 aligns well with the human EF-hand; however, the location of the second canonical EF hand in *Drosophila* LETM1 is unique to the fly (Figure 1 and Figure 3). A careful examination of *Drosophila* LETM1 function and how the second EF-hand regulates LETM1 function remains unsolved. Nevertheless, LETM1 function in both vertebrates and fly must be vital since deletion of *LETM1* leads to embryonic lethality in mice and *Drosophila* [59,69].

Mouse and human LETM1 orthologs share 83.8% global sequence identity and 93.2% sequence similarity as calculated with LALIGN global alignment similarity program [81]. Of note, the human ortholog contains a Pro20 insertion within the signal peptide sequence that is absent in the mouse. Likewise, human LETM2 and its mouse ortholog have high sequence identity and similarity at 77.2% and 87.2%, respectively (Figure 3). Both mouse and human LETM1 orthologs share evolutionarily conserved lysine acetylation of Lys597 and Lys596, respectively. Acetylation of lysine is a reversible post-translational modification (PTM) that is common in high pH and acyl-coenzyme A (acyl-CoA) environments, most certainly present in mitochondria. Mitochondrial proteins are extensively acetylated and have been shown to be catalyzed both non-enzymatically in vitro and enzymatically through acetyltransferase ACAT1 and deacetylase SIRT3 activity [82,83,84,85]. Lysine acetylation neutralizes the positive charge at the distal end of the residue; further, Lys acetylation can affect protein structure and interactions of coiled coil domains. Acetylated enzyme activity has been shown to be either increased or decreased; however, the majority of studies show acetylation generally decreases enzymatic activity [86]. How acetylation of Lys597 impacts LETM1 function remains unclear; however, pH changes and concentrations of acyl-CoA within the matrix represent important potential modulators of LETM1 protein structure and function requiring further study. 

## 3. The Role of LETM1 in Mitochondrial Physiology

### 3.1. LETM1 in K^+^/H^+^ Exchange

The role of LETM1 in mitochondrial physiology remains the focus of intensive research. The delicate balance of ions and gradients from the cytosol to matrix is a crucial determinant of health and disease. As mentioned above, cellular respiration generates an electrochemical proton gradient that polarizes the IMM, negatively charging the IMM and driving uptake of Ca^2+^ and K^+^ cations. Without extrusion of the subsequent accumulation of K^+^ within the matrix, osmotic swelling occurs leading to mitochondria dysfunction [32,69,70,77,87]. MDM38, the yeast LETM1 ortholog, was first proposed to be involved in KHE activity by Nowikowsky and co-workers [70] following studies focused on the effect of *MDM38* deletion (*MDM38Δ*) on yeast growth, mitochondria morphology and function. These studies demonstrated the apparent rescue of the petite phenotype, described as the decrease in cellular growth on non-fermentable substrates in *MDM38Δ* yeast strains [70,88,89]. Ablation of MDM38 was shown to reduce growth of W303 *Saccharomyces cerevisiae* yeast cultured with non-fermentable substrates, suggesting *MDM38Δ* resulted in a disruption in cellular respiration [70,89]. The use of nigericin, a selective K^+^ ionophore that facilitates electroneutral KHE and complementation with human LETM1 were independently shown to rescue *MDM38Δ* yeast cell growth, strongly suggesting yeast MDM38 and human LETM1 share the same underlying function. Furthermore, *MDM38Δ* in yeast cells has been shown to result in swelling and dissolution of well-defined cristae of the mitochondria, assessed by electron microscopy [70,89]. 

Other studies have similarly shown decreased cellular growth and mitochondrial swelling from LETM1 ortholog knockouts and knockdown including *Drosophila melanogaster* DmLETM1, *Caenorhabditis elegans* LETM1, human LETM1, and even LETM1 expressed in the divergent protozoan flagellate *trypanosoma brucei*, a member of the Kinetoplastea that contain a single mitochondrion [68,69,77,87]. In all of these studies performed in various organisms and cell cultures, again the use of nigericin, a selective K^+^ ionophore that allows electroneutral KHE, and complementation of LETM1 expression were shown to rescue both cell growth and mitochondria morphology. 

Mitochondria swelling is most often caused by K^+^/H^+^ imbalance. However, it remains possible that the swelling is caused by accumulation of other ions such as Ca^2+^ and magnesium (Mg^2+^) or biomolecules such as glutathione. Nigericin-mediated KHE activity may alleviate osmotic stress and KHE activity may compensate for non-K^+^ ion imbalance. Hypo-osmotic potassium acetate (KOAc) assays induce swelling in normal functioning mitochondria and represent a well characterized method to determine monovalent cation dysregulation [89,90]. KOAc is introduced to isolated mitochondria, and acidification of the matrix ensues due to an initial uptake of acetic acid. Subsequently, the external K^+^ is only taken up if there is KHE activity present, which then may lead to osmotic swelling. Deficiencies in KHE activity leads to the reduction in K^+^ uptake and subsequent reduction in swelling. Through the use of transcription switches mediating *MDM38Δ* and induction with quinine, a specific KHE inhibitor, KOAc assays offer compelling evidence MDM38, DmLETM1 and their orthologs are directly or indirectly involved in mediating KHE activity [69,70]. 

Submitochondrial particles (SMPs) are mitochondria that are exposed to ultrasound, which causes the cristae to pinch and invert the mitochondria, resulting in an inside out vesicle encompassing proteins normally found in the IMS and exposing proteins that normally face the matrix [91,92]. SMPs allow the study of the respiratory chain without complications from substrate transport and also allow further investigation into IMM ion transport. SMPs derived from wild-type and *MDM38Δ* DBY747 yeast were loaded with K^+^ and H^+^ selective fluorescent dyes (i.e., potassium-binding benzofuran isophthalate (PBFl) and proton-binding 2′,7′-bis-(2-carboxyethyl)-5-(and-6)-carboxyfluorescein (BCECF) to monitor the transport of K^+^ and H^+^ [92]. Froschauer and co-workers discovered through manipulation of the external and internal K^+^ and H^+^ concentrations that wild-type SMPs showed electroneutral antiporter KHE activity driven by the respective concentration gradients. Further, SMPs derived from *MDM38Δ* yeast showed reduced KHE activity which could be rescued with human LETM1 complementation [92]. A recent study utilizing the fluorescent K^+^ specific dye mitoPOP, which localizes within the matrix, showed siRNA-mediated LETM1 knockdown in HeLa cells increases the accumulation of K^+^ within the matrix compared to HeLa cells expressing wild-type LETM1 when incubated with KCl, supporting the aforementioned observations in yeast [88]. Collectively, the complementation by human LETM1 expression in cells with deleted LETM1 orthologs combined with the KOAc and fluorescent K^+^/H^+^ dye assays provide strong evidence that MDM38 and its human ortholog LETM1 are involved in regulating KHE activity. These data are supported by knockout studies in which ablated KHE activity lead to mitochondrial dysfunction [70,92]. 

### 3.2. LETM1 in Ca^2+^/H^+^ Exchange

The existence of a conserved canonical Ca^2+^ binding EF-hand motif in LETM1 from higher and lower order organisms, aside from the yeast MDM38 ortholog, suggests an involvement of Ca^2+^ in LETM1 function. More specifically, the fact that LETM1 contains a conserved EF-hand motif implies: (i) LETM1 exchanger activity directly requires Ca^2+^ binding to the EF-hand motif; (ii) the folding, structure and stability of LETM1 is dependent on Ca^2+^ binding in the EF-hand motif; or (iii) the EF-hand motif plays a role in buffering intracellular Ca^2+^ levels, separately from the exchanger activity of the molecule. The idea LETM1 was not involved in KHE but rather CHE activity of intracellular Ca^2+^ was first put forth by Jiang and co-workers in an RNA interference (RNAi) study performed in *D. melanogaster* cells searching for genes that affect intramitochondrial Ca^2+^ and H^+^ homeostasis [60]. A similar approach was used successfully to identify key molecules regulating store operated calcium entry (SOCE) [93,94,95,96,97]. Using the genetically encoded fluorescent pericam probe targeted to the mitochondria which reports Ca^2+^ concentration at one excitation wavelength (i.e., 405 nm) and H^+^ concentration at a second excitation wavelength (i.e., 488 nm), *Drosophila* S2 and HEK293 cells expressing LETM1 orthologs exhibited the uptake of Ca^2+^ into the matrix concomitant with an efflux of H^+^ out of the matrix [60]. In a Na^+^-free environment, Jiang et al. showed significant CHE antiporter activity, indicating a mechanism of Ca^2+^ uptake and extrusion dependent on pH and independent of NCLX mediated Na^+^/Ca^2+^ exchange. Furthermore, knockdown of DmLETM1 was shown to significantly abolish both pH-dependent Ca^2+^ transport and Ca^2+^-dependent pH changes [60]. Studies using human HEK293T and HeLa cell cultures with siRNA-mediated LETM1 knockdown showed similar decreases in pH-dependent Ca^2+^ uptake and extrusion using fluorescent mitochondrial Ca^2+^ indicators, GCaMP2-mt and dihydrorhod-2 AM (Rhod-2) [58,61]. Interestingly, cell lines overexpressing LETM1 harboring a deleted EF-hand or the D676A/D687K mutation within the Ca^2+^ binding EF-hand loop (i.e., loop residues 676–687; Figure 1 and Figure 3) as well as fibroblasts derived from WHS patients (see below) who are haploinsufficient in LETM1, all showed impaired Ca^2+^ transport. Reconstitution of wild-type LETM1 in cells expressing the LETM1 mutants or in cells with LETM1 knockdown rescued mitochondrial Ca^2+^ transport [58,61]. These results not only strongly suggest that LETM1 is involved in Ca^2+^ transport independent of NCLX, but also demonstrate that the EF-hand is a key motif required in some manner for LETM1 CHE function. 

**Figure 4:**
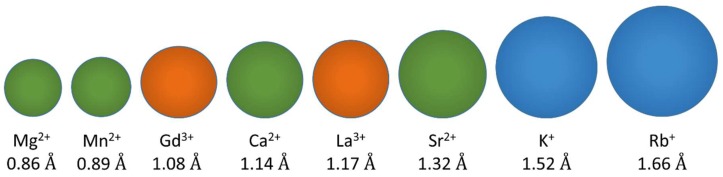
Relative sizes of selected monovalent, divalent and trivalent cations. Monovalent K^+^ and Rb^+^ shown in blue, divalent Ca^2+^, strontium (Sr^2+^), Mg^2+^, and Mn^2+^ in green, and trivalent gadolinium (Gd^3+^) and lanthanum (La^3+^) in orange. The sphere sizes scale with the relative ionic radii (indicated below each sphere). Ionic radii are from [100].

To understand the fundamental function of LETM1 in mitochondrial ion transport, recent studies have utilized in vitro proteoliposome assays to determine whether LETM1 directly transports Ca^2+^ or K^+^. Proteoliposomes containing LETM1 have shown rapid accumulation and extrusion of Ca^2+^ and not K^+^ indicating LETM1 may directly mediate Ca^2+^ transport [59,61,63]. In these proteoliposome assays, Ca^2+^ and K^+^ selective fluorophores and radioactive Ca^2+^ and rubidium (Rb^+^) isotopes are used to show that LETM1 reconstituted liposomes effectively uptake and extrude Ca^2+^ in the presence of an inward or outward Ca^2+^ gradient, respectively; further, Ca^2+^ transport is observed concomitant with pH changes, as LETM1 drives Ca^2+^ exchange for H^+^ and vice versa [59,61,63]. Note that Rb^+^, a faithful K^+^ analog, is used in lieu of K^+^ radioisotopes due to the longer half-life, and “inward gradient” is defined as a higher concentration of the ion outside relative to inside the compartment while “outward gradient” is defined as a lower concentration of the ion outside relative to inside the compartment. In similar experiments, LETM1 reconstituted proteoliposomes were shown to be unable to mediate the exchange of K^+^ or Rb^+^, in contrast to previous observations of LETM1-mediated KHE activity [63]. Ion selectivity experiments of LETM1 reconstituted in proteoliposomes suggested a strong affinity to Ca^2+^ and interference of Ca^2+^ transport by similar divalent cations and lanthanides, most notably manganese (Mn^2+^). Mn^2+^-sensitive fluorophores loaded in LETM1 reconstituted proteoliposomes showed rapid uptake and transport of Mn^2+^, which supports the notion LETM1 directly transports Ca^2+^, as Mn^2+^ is a similarly sized divalent cation that has been used as a faithful Ca^2+^ substitute by other Ca^2+^ transporters (Figure 4) [63,98,99]. 

LETM1 facilitated transport of Ca^2+^ in exchange for H^+^ is further supported by results that show outward or inward concentration gradients of Na^+^ and chloride (Cl^-^) have no effect on Ca^2+^ efflux; however, changes in pH have been shown to induce transport of Ca^2+^ in proteoliposomes with symmetrical Ca^2+^ concentrations (i.e., equal Ca^2+^ concentrations inside and outside of the liposome), consistent with the CHE activity [63]. The electrogenicity of proposed LETM1 CHE activity remains convoluted, however, as there are conflicting studies indicating LETM1 acts as a 1:1 electrogenic Ca^2+^/H^+^ and a 1:2 electroneutral Ca^2+^/2H^+^ exchanger [59,61,63]. Further assessments on the electrogenicity of LETM1 ion transport is required to tease out the precise ion stoichiometries involved in LETM1 function. 

Another ambiguous property of LETM1 concerns the inhibition by small molecules. Some studies indicate LETM1 can be inhibited by the MCU inhibitors ruthenium red (RR) and its analog ruthenium-360 (Ru360) as well as the NCLX inhibitor CPG-37157, while other studies show no inhibition of LETM1 by any of these compounds [60,63]. Nevertheless, in vitro studies using isolated mitochondria and reconstituted proteoliposomes offer strong support for the role of LETM1 in both influx and efflux of Ca^2+^ in exchange for H^+^. The fact that EF-hands are well conserved amongst LETM1 orthologs and the direct impact they have on mitochondrial Ca^2+^ transport as assessed via mutations and deletions showcase the importance of the EF-hand in LETM1 function and highlights a functionally critical area of LETM1 that remains structurally unresolved at high resolution.

### 3.3. LETM1 Protein Structure and Assembly

**Figure 5:**
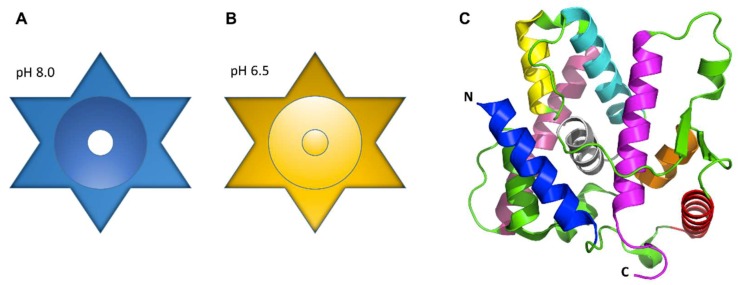
Three-dimensional architectures of human LETM1 and yeast MDM38. (**A**) The plan (top) view of the recombinant human LETM1 hexamer at pH 8.0 showing an open cavity at the center. (**B**) The plan (top) view of the recombinant human LETM1 hexamer at pH 6.5 showing the central cavity blocked at pH 6.5. (**C**) Ribbon representation of the backbone structure of the C-terminal MDM38 14-3-3-like domain spanning residues 182–408. The crystal structure was resolved at 2.1 Å. The human LETM1 architecture shown in (**A**,**B**) was determined by negative stain electron microscopy, and the shapes are interpretations of the low resolution EM structures shown by Shao and co-workers [61]. The structure in (**C**) was generated using the 3SKQ.pdb coordinates [102] in PyMOL.

In many organisms, the precise assembly state of LETM1 required for ion transport remains poorly understood, whether it be as a regulatory subunit in a heteromeric complex or as a homomeric channel. Proteoliposome studies of pure LETM1 displaying transporter activity suggests LETM1 oligomerizes into a homomeric exchanger. Various pulldown assays have described LETM1 forming complexes ranging from ~300 to 500–600 kDa [62,77,87,101]. It is important to note that the variability in apparent complex weight derived from these pulldown assays may be due to the use of different orthologs and pulldown techniques. Size exclusion chromatography analysis and sodium dodecyl sulfate polyacrylamide gel electrophoresis (SDS-PAGE) experiments showed that purified human LETM1 elutes under native solution conditions as a ~400 kDa complex, with a ~66–70 kDa monomer size, suggesting LETM1 forms a hexamer [61]. Circular dichroism analyses revealed significant secondary structure changes are caused by shifts in pH ranging from 6.5 to 7.0–8.0, showing decreased α-helicity below pH 7.0 [61]. Shao and co-workers further characterized the three-dimensional structures of purified human LETM1 using negative stain electron microscopy (EM) at pH 6.5 and 8.0. The two-dimensional class averages of the LETM1 particles observed by EM were consistent with a hexamer at both pH values; moreover, the subsequent three-dimensional reconstruction of the particles with an imposed six-fold symmetry, suggested that, in the absence of any accessory protein regulators, LETM1 forms a star shaped structure containing a central cavity of ~10.5 Å at pH 8.0, presumably for ion transport (Figure 5A). At pH 6.5, this cavity apparently becomes plugged (Figure 5B) [61]. The proteoliposome reconstitution assays along with the proposed structural models support the notion LETM1 homo-oligomerizes into an exchanger, through which ion transport is regulated by changes in pH. 

In higher resolution studies, the crystal structure of the soluble C-terminal portion of the yeast MDM38 (i.e., residues 160–408) has been solved at a resolution of 2.1 Å, revealing a 14-3-3-like fold composed of nine alpha helices (Figure 5C) [102]. This C-terminal region of MDM38 is proposed to function as a ribosome binding domain. Indeed, MDM38 was shown to pulldown ribosomes in vitro, offering support to studies showing reduced amounts of IMM proteins following *MDM38Δ* [89,102,103,104]. Additionally, Lupo and co-workers showed the LETM1 ion transport function was independent of ribosomal binding involved in mitochondrial translational regulation, showing that deletion of the ribosome binding domain and dysregulation of the translation of IMM proteins involved in electron chain transport are responsible for the decrease in respiring cell growth observed in *MDM38Δ* cells [102].

## 4. Wolf-Hirschhorn Syndrome (WHS) and other LETM1-Associated Pathophysiologies

WHS is a genetic disease most commonly arising from single copy de novo deletion of a portion of the short arm of chromosome 4 prior to birth. WHS is characterized by seizures, impaired cognitive function, congenital heart defects, facial abnormalities including a flat nose and enlarged forehead resulting in the so-called Greek warrior helmet appearance, and delayed growth and development [65,66]. Patients with WHS have weak muscle development and demonstrate a failure to gain weight, resulting in a short stature morphology. Facial abnormalities such as widely spaced eyes, underdeveloped jaws and defects of the eye and optic nerves represent severe quality of life impairments. Lingual processing and speech are notably impaired in WHS patients resulting in delayed development of communication skills. While some patients with WHS experience only some of these complications, the full WHS phenotype is further characterized by epileptic seizures that start at birth and often result in death [66,77]. 

A region spanning ~165 kilobases in chromosome 4 represents the region deleted in WHS patients, comprising *LETM1*, *MSX1*, a homeobox gene involved in the development of mouth and teeth structures, and *WHSC1*, a histone H3 lysine methyltransferase that interacts with β-catenin [65,66,67,101]. The precise molecular mechanisms of LETM1 involvement in the pathogenesis of WHS remains unclear. However, in mouse studies, it has been shown that LETM1 deletion causes similar facial deformities as observed in human WHS patients [59]. Additionally, as mentioned above, fibroblasts derived from WHS patients show decreased Ca^2+^ transport similar to cell cultures with LETM1 knockdown. Collectively, these studies indicate that LETM1 activity may be an important determinant in WHS, where a loss of LETM1-mediated CHE activity in neuronal mitochondria could lead to dysregulated Ca^2+^ homeostasis and signaling in individuals harboring the deletion in chromosome 4 [58,60,66].

LETM1 has been found to be overexpressed in breast, colon, esophagus, lung, ovarian, rectal, stomach, bladder and uterine cervical cancers [105]. This finding is supported by studies showing downregulation of LETM1 using siRNA results in reduced proliferation and invasion of T24 bladder cancer cells [106]. Consistently, LETM1 has also been shown to be upregulated in esophageal squamous cell carcinoma [107]. According to the catalogue of somatic mutations in cancer (COSMIC) database [108,109], there have been 183 somatic mutations in LETM1 associated with breast, liver, kidney, lung, prostate, hematopoietic and lymphoid tissue cancers. Interestingly, one mutation (i.e., E652K) results in a drastic charge swapping of an important residue in the canonical EF hand. E652 is located within the canonical EF-hand motif near the Ca^2+^ binding loop of human LETM1. Mechanistically, the substitution of acidic Glu for the basic Lys may affect the electrostatic guidance of Ca^2+^ into the EF-hand Ca^2+^ binding loop. Ultimately, a high resolution structure for LETM1 is required to unambiguously reveal the mechanisms whereby these 183 mutations may contribute to LETM1 dysfunction and various cancers.

## 5. Conclusions

Mitochondrial Ca^2+^ uptake not only influences cytosolic Ca^2+^ signals involved in myriad cellular processes, but also affects numerous mitochondrial processes integral to both cell survival and cell death. Several studies have shown LETM1 mediates a CHE activity in the mitochondria which is dependent on pH, thus indicating that LETM1 is integral to cytosolic and mitochondrial Ca^2+^ signaling. However, whether LETM1 directly or indirectly mediates CHE or KHE activity remains an important question in the mitochondrial biology field requiring more research. Associated with this question, LETM1 electrogenicity is incompletely understood. Regardless of the specific type of ion exchanger activity, LETM1 is undoubtedly vital for ion homeostasis within the mitochondrial matrix. 

High resolution structural information on LETM1 represents a major knowledge gap in the field and may unravel the precise ion exchange activity of LETM1. While a high-resolution structure of the assembled and functional LETM1 oligomer represents the idyllic goal, there are numerous specific structural questions which may be more simply obtainable and represent important structural targets: What mechanistic role does the canonical Ca^2+^ binding EF-hand play in LETM1 function? Does human LETM1 contain a second EF-hand motif, as in hundreds of other EF-hand proteins in nature, and what is the mechanistic role of this second EF-hand? How do post-translational modifications of LETM1 such as acetylation affect the structural mechanisms of exchanger function? How do point mutations in LETM1 associated with cancer affect the structure and function of LETM1? What are the structural mechanisms of LETM1 inhibition by RR, Ru-360 and CPG-37157, if any?

Collectively, the answers to these structural questions may provide insights into precisely how LETM1 functional deficiency contributes or leads to WHS. Importantly, LETM1 structural biology research will undoubtedly guide new therapeutic strategies, diagnostic tests and research tools which will help detect and treat WHS as well as other Ca^2+^ signaled malignancies.
